# Vibration Analysis of Vacancy Defected Graphene Sheets by Monte Carlo Based Finite Element Method

**DOI:** 10.3390/nano8070489

**Published:** 2018-07-02

**Authors:** Liu Chu, Jiajia Shi, Eduardo Souza de Cursi

**Affiliations:** 1 School of Transportation, Nantong University, Nantong 226019, China; chuliu@ntu.edu.cn; 2 Département Mécanique, Institut National des Sciences Appliquées de Rouen, 76801 Rouen, France; souza@insa-rouen.fr

**Keywords:** vacancy defects, Monte Carlo based finite element method, natural frequencies

## Abstract

The stochastic distributed placement of vacancy defects has evident effects on graphene mechanical property, which is a crucial and challenged issue in the field of nanomaterial. Different from the molecular dynamic theory and continuum mechanics theory, the Monte Carlo based finite element method (MC-FEM) was proposed and performed to simulate vibration behavior of vacancy defected graphene. Based on the Monte Carlo simulation, the difficulties in random distributed location of vacancy defects were well overcome. The beam element was chosen to represent the exact atomic lattice of the graphene. The results of MC-FEM have a satisfied agreement with that in the reported references. The natural frequencies in the certain vibration mode were captured to observe the mechanical property of vacancy defected graphene sheets. The discussion about the parameters corresponding with geometry and material property was accomplished by probability theory and mathematical statistics.

## 1. Introduction

For the parameters corresponding to material properties of defect-free graphene monolayer, Young’s modulus and intrinsic strength are 1.0 TPa and 130 GPa [1]. Large deviation in simulations and experiments has been found, which is attributed to the presence and uncertainty of defects in the nanotube structure [2,3]. Various complicated defects exist in the graphene sheet, and some defects in the atomic structure can even deteriorate the mechanical and electronic properties of graphene materials [4]. The research of vacancy defects in graphene sheets is very necessary, by which the uncertainties and fluctuation in mechanical property of graphene sheets can be reasonably explained and comprehensively understood.

Graphene, as a two-dimensional structure, was introduced in 2004 [5]. The main tough task in the analysis of the mechanical behavior of graphene is its small size [6,7,8,9]. Obviously, it is difficult to conduct physical experiments in nanoscale. Analytical and numerical methods are available alternatives in nanomaterial research. These methods can be divided into two categories: atomistic-based methods and continuum mechanics theories. Atomistic-based methods include the molecular dynamics simulation (MD) [10], tight-binding molecular dynamics [11] and density function theory [12]. Some size dependent non-classical continuum theories have been studied in the research field of graphene sheets, which includes the strain gradient theory [13], modified couple stress theory [14], and nonlocal elasticity theory [15]. However, atomistic based methods are computationally expensive when the amount of atoms in the system is large. In continuum theories, the defects or uncertainties in the nanostructure are difficult to be taken into consideration. According to the overview of the existing literature, it can be found that the influence of the vacancy defects on the mechanical behavior of nanostructures has been studied in very few papers.

Attempts and struggles for a deeper understanding of the influence of defects in graphene sheets have been undertaken in this challenging field. For instance, the effect of vacancy defects on Young’s modulus of graphene sheets was discussed by molecular dynamics [16]. The Stone–Wales defects in tensile behavior of graphene sheets and carbon nanotubes were accomplished by the same method [17]. In addition, molecular dynamics simulations are applied in studying mechanical property of defective single-layered graphene sheets [18]. The non-local elastic theory is also applied in vibration analysis of defective graphene sheets. However, the effects of structural defects on the vibration behavior of graphene sheets has rarely been investigated [19].

The motivation of this study is to effectively analyze vibration behavior of defected graphene sheets when the vacancy defects are randomly distributed. Since the physical experiments of vacancy defected graphene sheets are difficult and expensive, numerical simulation methods are another promising supplement that need to be developed. Besides, it is necessary to find an effective index to express and observe the effects of vacancy defects in vibration behavior of graphene sheets.

In the free vibration, natural frequencies of structures are the essential parameters, which are directly corresponding with the geometrical and material characteristics. The change of the vacancy distribution and location can cause fluctuation in natural frequencies of graphene sheets. The natural frequencies of vibration modes are very sensitive to the geometrical defects. Therefore, natural frequency is a good indicator to discuss the effects of vacancy defects in the graphene sheets.

Combining the Monte Carlo simulation with the finite element method to effectively simulate the random dispersed vacancy defects in graphene sheets is a perspective and feasible method. The Monte Carlo simulation as a sophisticated sampling method has been widely applied in the research of phase transition and magnetism of graphene sheets [20,21,22,23]. When the number of sampling is sufficient, it can reach a satisfied accuracy in numerical computations. It is common to take the results of the Monte Carlo simulation as the exact solution or comparison standard [24,25]. By combining the Monte Carlo method with the finite element model, an effective and feasible method is proposed in this paper. The difficulties in placement uncertainty, irregularity, and stochastic location are solved in the Monte Carlo simulation. The beam finite element model is well represented the specific hexagon microstructure of graphene sheets.

The remaining part of the paper is organized as follows: a clear introduction about the beam finite element model for representing graphene hexagon microstructure is presented. The Timoshenko beam theory for free vibration analysis is explicitly derived. Besides, the implementation of Monte Carlo based finite element method is expressed in computational program after the validation of deterministic beam finite element model. Good agreement is reached in the present model and reported literatures [26,27,28,29,30,31,32,33,34] for the vibration analysis of graphene sheets. The discussion extensively consists of the effect of amount of vacancy defects and geometrical and material parameters in the natural frequencies of vacancy defected graphene sheets. A short conclusion of this paper is provided.

## 2. Materials and Methods

### 2.1. Graphene Sheets

**Figure 1:**
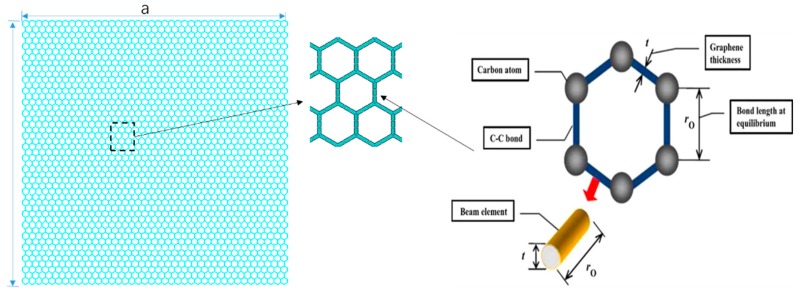
Schematic of a monolayer graphene.

Carbon atoms in graphene are bonded together with covalent bonds forming a hexagonal 2D lattice. The parameters to describe the bonds are the bond length and bond angle. The displacement of individual atoms under external forces is constrained by the bonds. In other words, the mechanical deformation of graphene sheets is the result of the interactions between the bonds. Graphene sheets are supposed as planar-frame structures, the bonds act as connecting load-carrying elements, and atoms are assumed as joints of the connecting elements. Then, classical structural mechanics methods such as the finite element method can be applied in the vibration analysis. Figure 1 depicts planar-frame model of graphene sheets by simulating the constitutional element carbon atoms as nodes and C–C action as linking beams. The entire graphene sheet’s lattice is modeled by periodic and regular hexagons.

The elastic geometrical properties of the beam elements have been derived using an energy relationship between molecular mechanics and continuum mechanics developed in [35]. According to the computation, the diameter d, Young’s modulus E, and shear modulus G of the beam elements representing the C–C bonds are derived from

(1) $$\left\{ \begin{array}{l} d=4\sqrt{\frac{k_{\theta }}{k_{r}}}\\ E=\frac{k_{r}^{2}L}{4\pi k_{\theta }}\\ G=\frac{k_{r}^{2}k_{\tau }L}{8\pi k_{\theta }^{2}} \end{array}\right.$$

where $k_{r},\;k_{\theta },\;k_{\tau }$ are the bond sketching, bond bending and torsional resistance force constants, respectively. In the finite element model of graphene sheets, the length of C–C bond is corresponding to the length of beam in planar-frame structure, wall thickness is related with the diameter of circular solid cross section of the beam elements, as in Figure 1.

The numerical simulation model of graphene sheets was created by the ANSYS parameter design language. For the modeling of the C–C bonds, the three-dimensional (3D) elastic BEAM188 element was used [20]. BEAM188 is suitable for analyzing slender to moderately stubby/thick beam structures. The element is based on the Timoshenko beam theory which includes first-order shear-deformation effects. The element is a linear, quadratic, and cubic two-node beam element in 3D. For each node, it has six degrees of freedom, which include translations in the *x*, *y*, and *z* directions and rotations around the *x*, *y*, and *z* axes. BEAM 188 is well-suited for linear, large rotation, and/or large strain nonlinear applications.

### 2.2. Finite Element Model in Vibration Analysis

The beam elements applied in graphene sheets are based on the Timoshenko beam theory, which is a first order shear deformation theory. In this theory, the transverse shear strain is constant through the cross section, which remains plane and undistorted after deformation.

The equation derived by Timoshenko that governs flexural vibrations of beams with constant cross section can be expressed as [36]

(2) $$\frac{EI}{\rho A}\frac{\partial^{4}\xi }{\partial z^{4}}-\frac{I}{A}(1+\frac{E}{\kappa G})\frac{\partial^{4}\xi }{\partial z^{2}\partial t^{2}}+\frac{\partial^{2}\xi }{\partial t^{2}}+\frac{\rho I}{\kappa GA}\frac{\partial^{4}\xi }{\partial z^{4}}=0$$

where ξ=ξ(z,t) is the transversal displacement along the *x*-axis at point *z* and time *t*, and *E* is the Young’s modulus, I is the inertia moment, *G* is the shear modulus, ρ is the mass density, and A is the cross section area. In this theory, the Timoshenko shear coefficient κ is a free parameter.

Besides ξ, an angular variable θ is introduced. During flexural motion, cross sections are supposed to remain flat and perpendicular to the deflected neutral axis at any point of this axis. The angle θ between the *z*-axis and a vector orthogonal to the cross section is equal to the angle between the neutral axis tangent line and the *z*-axis. Note that θ equals the slope of the deflected neutral axis, that is

(3) $$\theta \approx \tan\theta =\frac{\partial \xi }{\partial z}$$

In a normal mode, ξ(z,t) varies harmonically with time as

(4) ξ(z,t)=[Acos(wt)+Bsin(wt)]χ(z)=Csin(wt+φ)χ(z)

where *A*, *B*, *C*, and φ are the corresponding constants to be determined.w=2πf is the angular frequency and χ(z) is a function that determines the normal mode amplitude. Substituting this form of ξ into Equation (1),

(5) $$\frac{\partial^{4}\chi }{\partial z^{4}}+\frac{\rho w^{2}}{M_{r}}\frac{\partial^{2}\chi }{\partial z^{2}}+\frac{w^{2}\rho^{2}}{\kappa GE}[w^{2}-w_{c}^{2}]\chi =0$$

With $w_{c}=2\pi f_{c}=\sqrt{\frac{\kappa GA}{\rho I}}$, where $f_{c}$ is the critical frequency and $\frac{1}{M_{r}}=(\frac{1}{E}+\frac{1}{\kappa G})$ is the reduced modulus.

It is well known that solutions of the above equation behave differently according to $w^{2}-w_{c}^{2}$. The general solution can be written as

(6) $$\left\{ \begin{array}{ll} \chi (z)=A_{1}\sin(K_{1}z)+B_{1}\cos(K_{1}z)+C_{1}e^{K_{2}z}+D_{1}e^{-K_{2}z} & w<w_{c}\\ \chi (z)=A_{2}\sin(K_{1}z)+B_{2}\cos(K_{1}z)+C_{2}\sin(K_{2}z)+D_{2}\cos(K_{2}z) & w>w_{c} \end{array}\right.$$

where

(7) $$\left\{ \begin{array}{l} K_{1}=\sqrt{\frac{\rho w^{2}}{2M_{r}}+\sqrt{{\left(\frac{\rho w^{2}}{2M_{r}}\right)}^{2}-\frac{\rho^{2}w^{2}}{\kappa GE}(w^{2}-w_{c}^{2})}}\\ K_{2}=\sqrt{S\left[\frac{\rho w^{2}}{2M_{r}}-\sqrt{{\left(\frac{\rho w^{2}}{2M_{r}}\right)}^{2}-\frac{\rho^{2}w^{2}}{\kappa GE}(w^{2}-w_{c}^{2})}\right]} \end{array}\right.\;With\;S=\left\{ \begin{array}{ll} 1 & if\;w>w_{c}\\ -1 & if\;w<w_{c} \end{array}\right.$$

Usually, coefficients $A_{i},\;B_{i},\;C_{i},\;D_{i}$ are different from zero, and the solutions of equations include functions depending on both $K_{1}$ and $K_{2}$. Where $K_{1}$ and $K_{2}$ are defined as positive square roots.

For free vibration analysis for Timoshenko beam, based on principal of virtual work, the weak form of equation can be written as [36]

(8) $$\int_{0}^{L}EI\frac{\partial \theta }{\partial x}\delta (\frac{\partial \theta }{\partial x})dx+\int_{0}^{L}\kappa GA(\frac{\partial \xi }{\partial x}-\theta )\delta (\frac{\partial \xi }{\partial x}-\theta )dx=\int_{0}^{L}\delta \xi \rho A\ddot{\xi }dx+\int_{0}^{L}\delta \theta \rho I\ddot{\theta }dx$$

As defined above, ξ is the transversal displacement in Timoshenko beam, where θ is the transversal rotation, while $\ddot{\xi }$ and $\ddot{\theta }$ are the transverse and rotary accelerations, respectively, L is the length of the beam and δ denotes that the terms are virtual.

For free vibration

(9) $$\mathrm{Ku}-M\ddot{u}=0$$

where K and M are the global stiffness and mass matrices which contain contributions from element stiffness and mass matrices.

A general solution can be expressed as

(10) $$u=\phi_{k}e^{iw_{k}}$$

Substituting into Equation (8) yields

(11) $$[K-w_{k}^{2}M]\phi_{k}=0$$

where $\phi_{k}$ is a set of displacement-type amplitude at the control points otherwise known as the model vector, $w_{k}$ is the natural frequency associated with the *k*th mode.

This is an eigenvalue problem and for non-zero solutions the determinant of the equation must be zero

(12) $$\left|K-w_{k}^{2}M\right|=0$$

The discretization of governing equation can be done by unidirectional linear finite element with two nodes. In each axes direction, there are two degrees of freedom at each node, and six degrees of freedom for each node in total. The approximated solution in the displacement field, transversal displacement and rotation, can be written as [37]

(13) $$\left\{ \begin{array}{l} \xi =\sum_{i=1}\varphi_{i}\xi_{i}\\ \theta =\sum_{i=1}\varphi_{i}\theta_{i} \end{array}\right.$$

Consider the natural coordinate ς=[−1, 1] in the element domain,

(14) $$\begin{array}{l} \xi (\varsigma )=\varphi_{1}(\varsigma )\xi_{1}+\varphi_{2}(\varsigma )\xi_{2}\\ \theta (\varsigma )=\varphi_{1}(\varsigma )\theta_{1}+\varphi_{2}(\varsigma )\theta_{2} \end{array}$$

In matrix form, it can be presented by

(15) $$\left\{\begin{array}{l} \xi (\varsigma )\\ \theta (\varsigma ) \end{array}\right\}=[H]\left\{u\right\}$$

where [H] is the matrix of shape functions and {u} is vector of nodal displacements.

(16) $$[H]=\left[\begin{array}{cc} \begin{array}{ccc} \varphi_{1} & 0 & \varphi_{2} \end{array} & 0\\ \begin{array}{ccc} 0 & \varphi_{1} & 0 \end{array} & \varphi_{2} \end{array}\right],\;\left\{u\right\}=\left\{\begin{array}{c} \xi_{1}\\ \theta_{1}\\ \xi_{2}\\ \theta_{2} \end{array}\right\}$$

By substituting to the weak form of equation, the stiffness and mass matrix in element domain are written as

(17) $$\begin{array}{l} {}[K^{e}]=\int_{-1}^{1}{\left[B\right]}^{T}\left[D\right]\left[B\right]\left|J\right|d\varsigma \\ {}[M^{e}]=\int_{-1}^{1}\rho I{\left[H\right]}^{T}\left[H\right]\left|J\right|d\varsigma +\int_{-1}^{1}\rho A{\left[H\right]}^{T}\left[H\right]\left|J\right|d\varsigma \end{array}$$

where [B] is the strain displacement matrix, [D] is the constitutive matrix, and |J| is the Jacobian determinant.

(18) $$\begin{array}{l} \left[B\right]=\left[\begin{array}{cc} \begin{array}{ccc} 0 & \frac{1}{\left|J\right|}\left(\frac{d\varphi_{1}}{d\varsigma }\right) & 0 \end{array} & \frac{1}{\left|J\right|}\left(\frac{d\varphi_{2}}{d\varsigma }\right)\\ \begin{array}{ccc} \frac{1}{\left|J\right|}\left(\frac{d\varphi_{1}}{d\varsigma }\right) & -\varphi_{1} & \frac{1}{\left|J\right|}\left(\frac{d\varphi_{2}}{d\varsigma }\right) \end{array} & -\varphi_{2} \end{array}\right]\\ \left[D\right]=\left[\begin{array}{cc} EI & 0\\ 0 & \kappa GA \end{array}\right] \end{array}$$

For large symmetric eigenvalue problems, the Block Lanczos eigenvalue extraction method is popular and available [38]. A fast convergence rate can be reached by this method. In modal analysis, Block Lanczos method is combined with Sturm sequence checks, the number of eigenvalues requested is extracted by an automated shift strategy. In addition, the Sturm sequence check also makes sure that the requested number of eigen-frequencies beyond the user supplied shift frequency is found without missing any modes [39].

### 2.3. Monte Carlo Based Finite Element Method

The Monte Carlo approach is a method developed to compute integrals by random number generation. Suppose a complex integral like

(19) $$F=\int_{a}^{b}h(x)dx$$

Decompose h(x) into the production of a function f(x) and a probability density function p(x) defined in the interval (a, b), then the integral can be written as

(20) $$F=\int_{a}^{b}h(x)dx=\int_{a}^{b}f(x)p(x)dx=E_{P(x)}\left[f(x)\right]$$

If a large number of random variables from the probability density p(x) are sampling, then

(21) $$F=\int_{a}^{b}h(x)dx=E_{P(x)}\left[f(x)\right]≃\frac{1}{n}\sum_{i=1}^{n}f(x_{i})$$

Consider the integral I(y)=∫f(y|x)p(x)dx, it can be approximated by Monte Carlo simulation as

(22) $$\hat{I}(y)=\frac{1}{n}\sum_{i=1}^{n}f(y|x_{i})$$

Therefore, the estimated Monte Carlo standard error can be expressed as

(23) $$\varepsilon^{2}=\frac{1}{n}\left(\frac{1}{n-1}\sum_{i=1}^{n}\left(f(y|x_{i}\right)-\hat{I}(y){)}^{2}\right)$$

Based on Equation (23), when n is amplified, the Monte Carlo standard error will become tiny, which means that the Monte Carlo simulation can reach a satisfied accuracy when the number of sampling is large enough. However, the huge sampling sets for the finite element model cost expensive computation. There is a trade-off between the results’ accuracy and computational time costs. Depending on the work of CHU in 2015 [24], the number of Monte Carlo simulation is chosen as 500 in this study.

**Figure 2:**
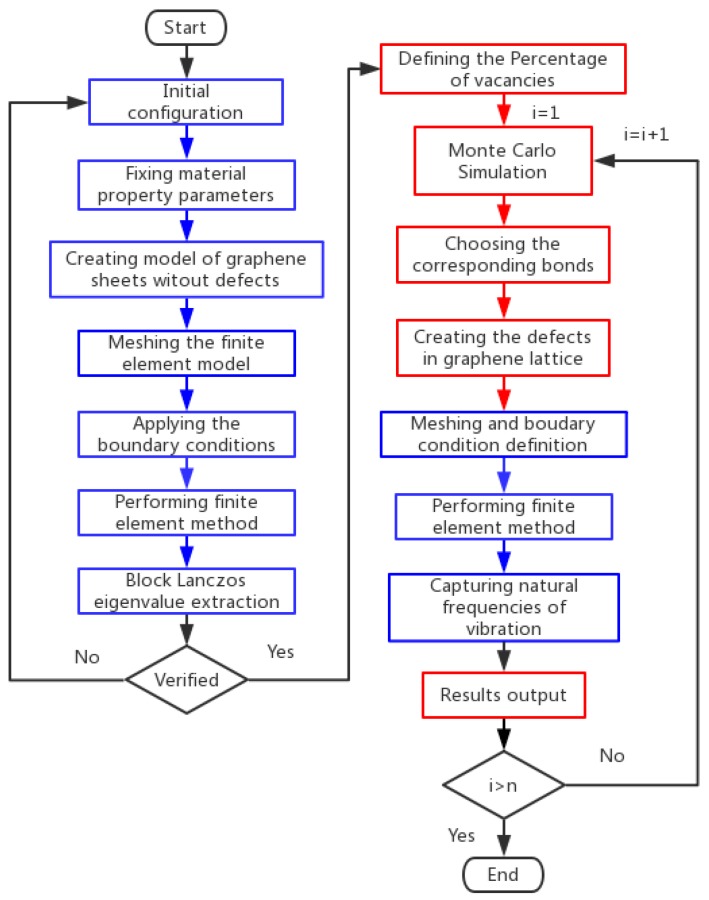
Flowchart of Monte Carlo based finite element method.

In order to implement the Monte Carlo based finite element method for vibration analysis of graphene sheets, the flowchart is present in Figure 2. The blue boxes represent the program of the finite element method, the red boxes indicate the Monte Carlo simulation.

In order to combine the Monte Carlo simulation with the beam finite element method, the exact design of the computational simulation in the flowchart can be concluded in seven steps:

Step 1: Define the initial configuration of graphene sheets, which includes corresponding parameters of bonds’ length, height, and width of a hexagonal 2D lattice, and also thickness or diameter of the section.
Step 2: Fix material property parameters, which consist of the Young’s modulus, Poisson ratio, physical density. The accuracy of the finite element model for graphene sheets heavily depends on exact parameters of material property.
Step 3: Mesh the beam elements and apply the boundary conditions, which is a traditional step of the finite element method.
Step 4: Perform the finite element method and use the Block Lanczos eigenvalue extraction for natural frequencies in each mode vibration of graphene sheets. If the deterministic finite element model is confirmed to be validated, the Monte Carlo simulation will commence, otherwise, return back to the step of initial configuration.
Step 5: Apply the Monte Carlo simulation method to randomly disperse vacancy defects in the graphene sheets in the certain percentage. The corresponding number of chosen bonds is evident and can be used to create the vacancy defects in the graphene.
Step 6: Mesh the beam elements and apply the boundary conditions, and perform the finite element model computation.
Step 7: Capture the natural frequencies of graphene with vacancy defects in the vibration mode and output the results.

Taking consideration of the effects of stochastic distributed vacancy, the loop needs to repeat to provide reliable and accurate output results. The loop continues until the Monte Carlo simulation is completed.

## 3. Validation of the Model

By commercial software of ANSYS (Version 14.5, APDL, Cannonsburg, PA, USA), the typical graphene sheet lattice structure is created, which is in accord with the certain sizes in the literatures. In the truss finite element model, there are 6226 trusses, 4212 points and 16,664 nodes created. Performing the beam finite element model for graphene without vacancy defects, a good agreement between the present results and that of relevant references is achieved. Table 1 lists the corresponding parameters of material property of graphene in literatures, and in proposed model, the results of natural frequencies for the first four modes of vibration.

**Figure 3:**
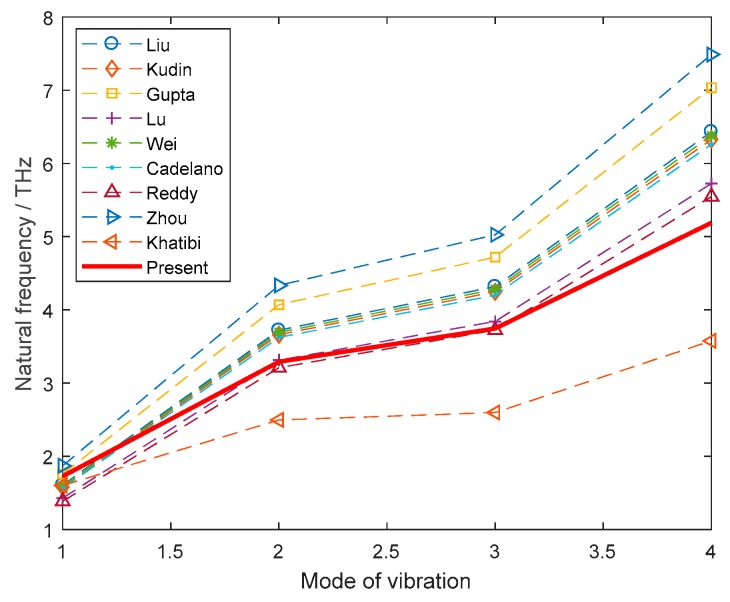
Comparison natural frequencies of present model with that in literatures.

From Table 1 and Figure 3, it is obvious that the present results of the beam finite element model have satisfied agreement with the corresponding results in the reported references. To be more exact, the natural frequencies of graphene in this study are close to the results of Reddy, Lu, Wei, Kudin, Liu, and Cadelano; slightly higher than the results of the molecular dynamics used by Khatibi; and lower than the results of Zhou and Gupta. The present beam finite element model is feasible to be applied in the subsequent study of vacancy defected graphene sheets.

The vacancy defects are quantified according to defect density, which is defined as

(24) $$Per=\frac{D_{n}}{A_{n}}$$

where $D_{n}$ is the amount of vacancy defects and $A_{n}$ is the total quantity of beams in graphene.

The relative error is used to calculate the difference of the mean of the natural frequencies with that of graphene without defects. Error can be written as

(25) $$Error=abs(P_{f}-M_{f})$$

where $M_{f}$ is the mean of natural frequencies of vacancy defected graphene, $P_{f}$ is the natural frequency of pristine graphene.

**Figure 4:**
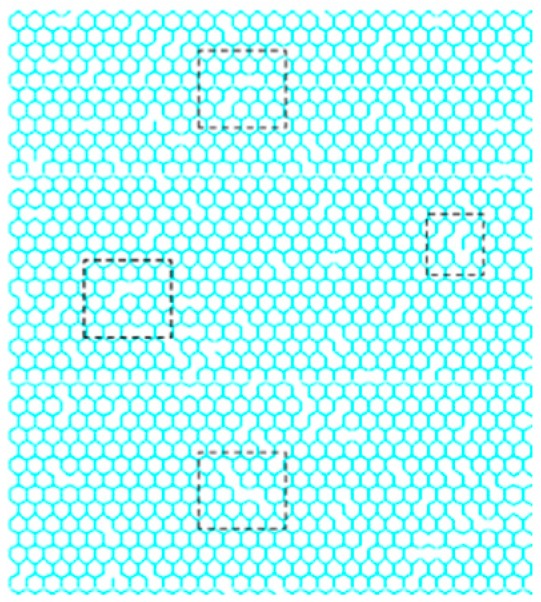
Randomly distributed vacancy in graphene sheet by Monte Carlo simulation.

Figure 4 depicts a possible example for the Monte Carlo based stochastic distributed placement of vacancy in graphene sheets.

## 4. Results and Discussion

In the following part, the explicit discussion about the amount of vacancy defects and parameters corresponding to geometrical and material characteristics of graphene sheets are sequentially presented. The results are computed by the Monte Carlo based finite element method and demonstrated by the probability theory and mathematical statistics.

### 4.1. Amount of Vacancy Defects

The amount of vacancy defects accounted in this study is by the absence of beam in the stochastic placement. Table 2 provides the statistical results of the first four order natural frequencies of vibration modes for graphene sheets with different number of vacancy defects. By taking the uncertainty of randomly distributed location of vacancy defects into consideration, the probability and statistical results of natural frequencies are more reliable and suitable for the study of vibration behavior of graphene sheets. It is clear that, with the increase of vacancy defects, the mean of the first four order natural frequencies are diminished. When *Per* is 0.5% and 1%, the changes of natural frequencies caused by randomly distributed vacancy defects are not evident. However, the reduction of natural frequencies are abrupt when the percentage of vacancy defects exceeds 1%.

**Figure 5:**
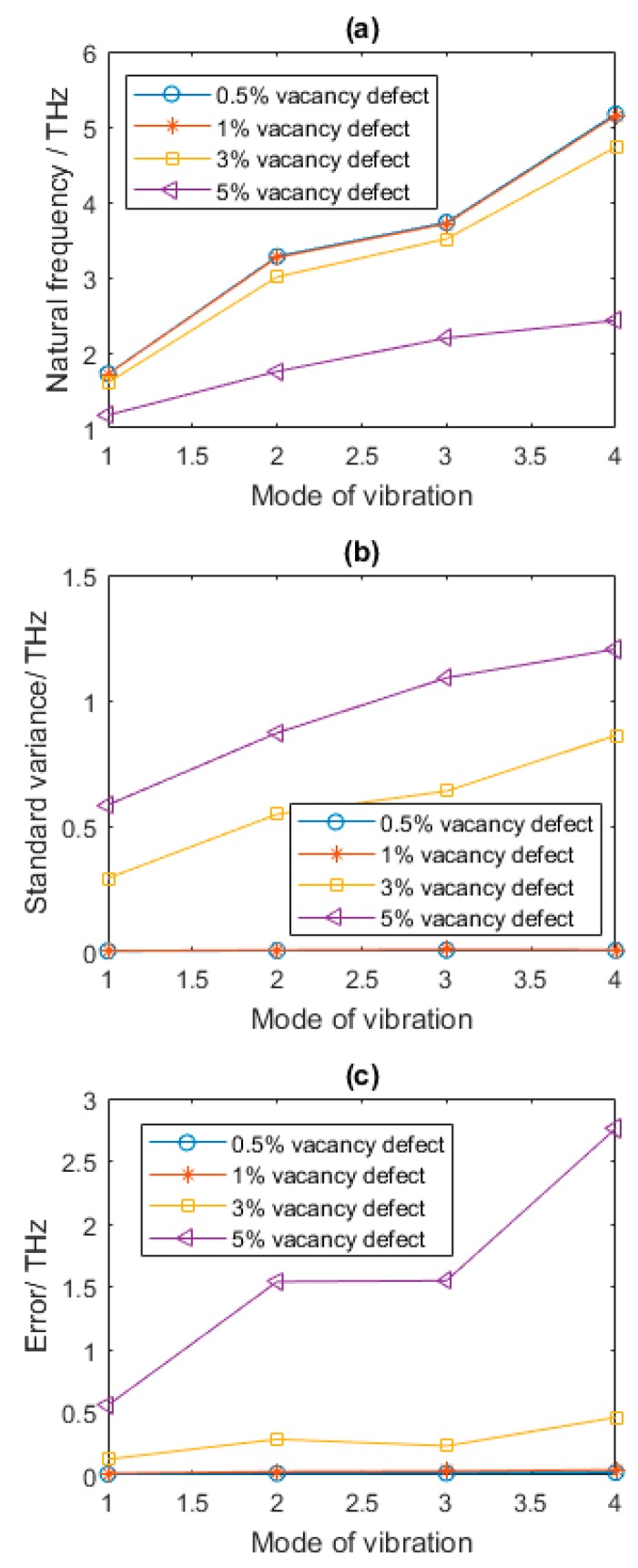
Results of defected graphene sheets with different amount of vacancy defects (**a**–**c** for mean, standard variance and Error of natural frequency, respectively).

The natural frequencies of the beam finite element model as derived in Equation (9) are corresponding to the stiffness and mass matrices. The fluctuation in natural frequencies in different vibration modes is a comprehensive effects of change in Young’s modulus and mass reduction by vacancy defects. According to the relative reference [40], the elastic modulus of graphene can increase by well-controlled defect creation. The Young’s modulus reaches at the peak when the percentage of vacancy defects of carbon atoms is 0.2%. It is reasonable to find that when Per equals to 0.5% and 1%, the changes of natural frequencies (Error) in Table 2 and Figure 5c are not evident. 

When Per is 3%, the reduction of the first four order natural frequencies compared with that of pristine graphene are 7.1%, 8.7%, 6.04%, and 8.7%, respectively. Besides, the reduction reaches 32.1%, 46.7%, 41.3%, and 53.1% for first four order vibration mode when Per equals to 5%. As shown in Table 2 and Figure 5, with the increase of vacancy defects, the first four natural frequencies all have different degrees of reduction. Besides, the standard variance of natural frequencies becomes larger along with the increase of Per. Furthermore, in the first four vibration modes, the standard variances increase sharply when Per is above 1%. The vacancy defects greatly affect the vibration behavior of graphene sheets. The existence of vacancy defects changes the structure of graphene lattice and has a profound impact on natural frequencies.

As mentioned above, natural frequencies are the ratios of stiffness and mass matrices. The appearance of vacancy defects influences both the stiffness and mass matrices. The mass of graphene sheets decreases according to the increase of the amount of vacancy defects. The influence of vacancy defects in Young’s modulus of graphene sheets is not monotonous and non-linear [31,41]. When the number of vacancy defects is small, by effectively controlling the distribution of vacancy defects, the augmentation of the amount of vacancy defects in graphene sheets can increase the Young’s modulus [40]. However, when the amount of vacancy defects reaches a certain value, the Young’s modulus dramatically reduces. The augmentation of vacancy defects leads to not only cutting down in Young’s modulus, tensile strength, and failure strain, but also fracture propagation and crack formation [42]. Vibration analysis is an appropriate and feasible way to observe the influence of the amount of vacancy defects in graphene sheets.

### 4.2. Geometrical Parameters

On the basis of molecular mechanics by the energy approach, the tube diameter and length strongly effect the material properties, such as Young’s modulus, shear modulus, bend-angle and strain energy of single- and multi-walled nanotubes [43]. The discussion about geometrical parameters, namely bond length *L*, diameter of cross section *D*, and the amount of hexagons in *y* axis *N*, is important and necessary in vibration analysis of graphene sheets.

**Figure 6:**
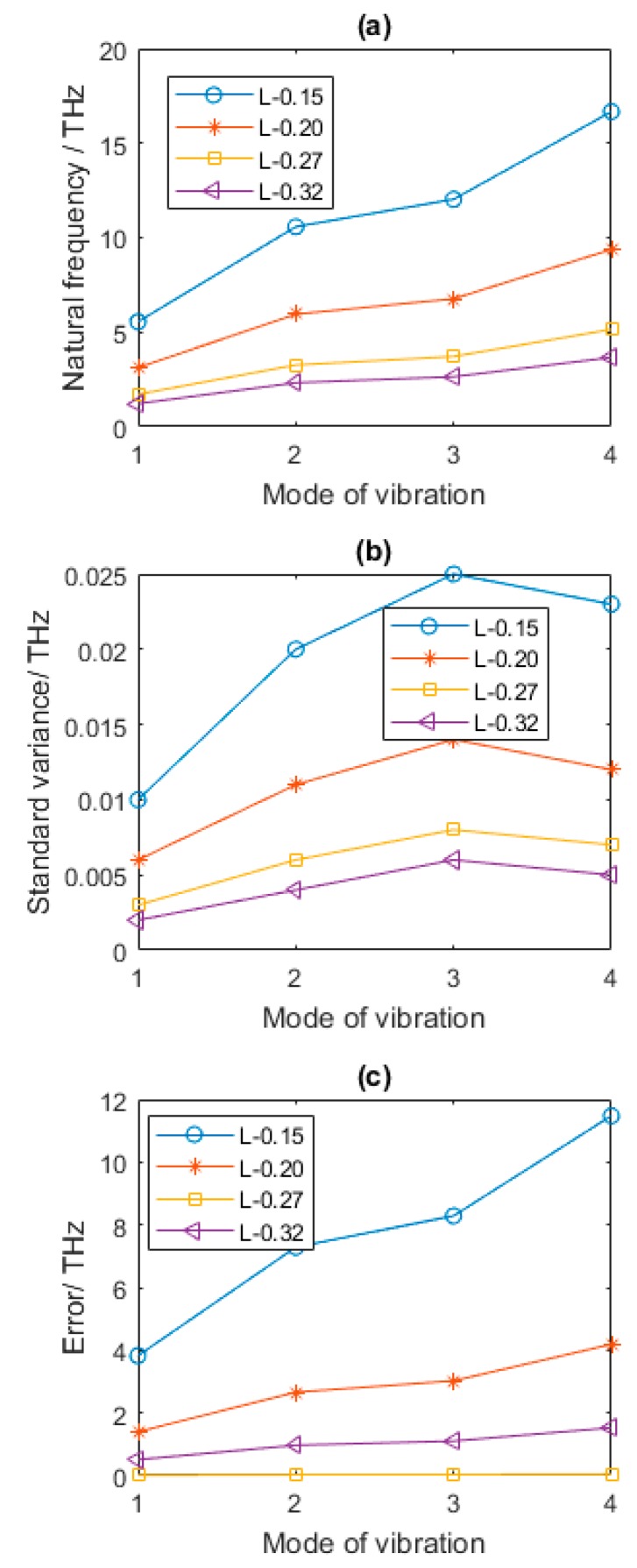
Results of defected graphene sheets with different *L* (**a**–**c** for mean, standard variance and Error of natural frequency, respectively).

The results are based on the statistical results by consideration of the random dispersion of vacancy location in the graphene sheets. In Figure 6, with the amplification of *L*, the mean of first four order natural frequencies is distinctly cut down. The standard variance and error in Figure 6b,c also follow this rule. When the amount of the vacancy defects is 1% and *L* is 0.15, with the augmentation of length of each bond in hexagon of graphene sheets, the increase of the first four order natural frequencies compared with that of graphene sheets in Table 2 are 223.98%, 224.01%, 224.00%, and 223.90%, respectively. When *L* is equal to 0.2, the increases are 82.26%, 82.27%, 82.25%, and 82.22%, respectively. When *L* is 0.32, the reductions of the first four order natural frequencies of vibration modes are 28.82%, 28.79%, 28.82%, and 28.82%, respectively. Therefore, the length of bond in hexagon of graphene sheets is a crucial factor, which shows a good agreement of size effects in physical experiments and numerical simulation.

**Figure 7:**
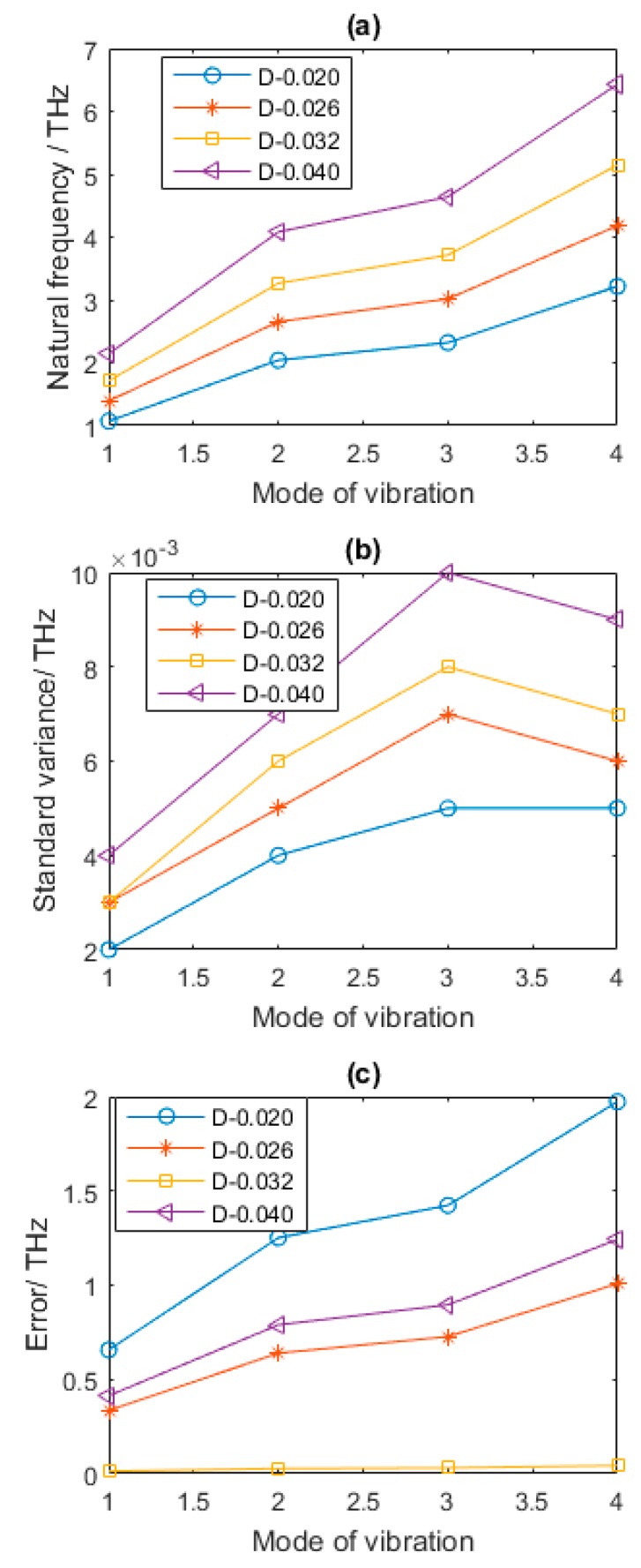
Statistical results of defected graphene sheets with different *D* (**a**–**c** for mean, standard variance and Error of natural frequency, respectively).

According to the results above, the geometrical parameter *L* has a negative effect on first four order natural *frequencies*. The larger *L* is, the smaller the natural frequencies are. Different from the effects of *L* in vibration behavior of vacancy defected graphene sheets, with the increase of *D*, the first four order natural frequencies are amplified. Figure 7 well proves this point. The standard variance in Figure 7b also follows this rule, but the results of errors in Figure 7c are different. Compared with the results in Table 2 when Per equals to 1% and *D* is 0.02, the reduction of the first four order natural frequencies of vibration modes are 37.51%, 37.49%, 37.49%, and 37.50%, respectively. When *D* is equal to 0.026, the reduction of the first four order natural frequencies of vibration modes compared with that of graphene in Table 2 are 18.73%, 18.74%, 18.74%, and 18.75%, respectively. When *L* is 0.04, the increases of the first four order natural frequencies of vibration modes are 24.97%, 25.02%, 24.97%, and 24.99%, respectively. In Equations (2) and (8), both the cross section and inertia moment are related with the diameter of cross section *D*, which has more complicated effects in natural frequencies of vibration modes. Even though the change of extent in natural frequencies is not as large as the effect of the bond length *L*, more emphasis should be put on the diameter of cross section *D*.

**Figure 8:**
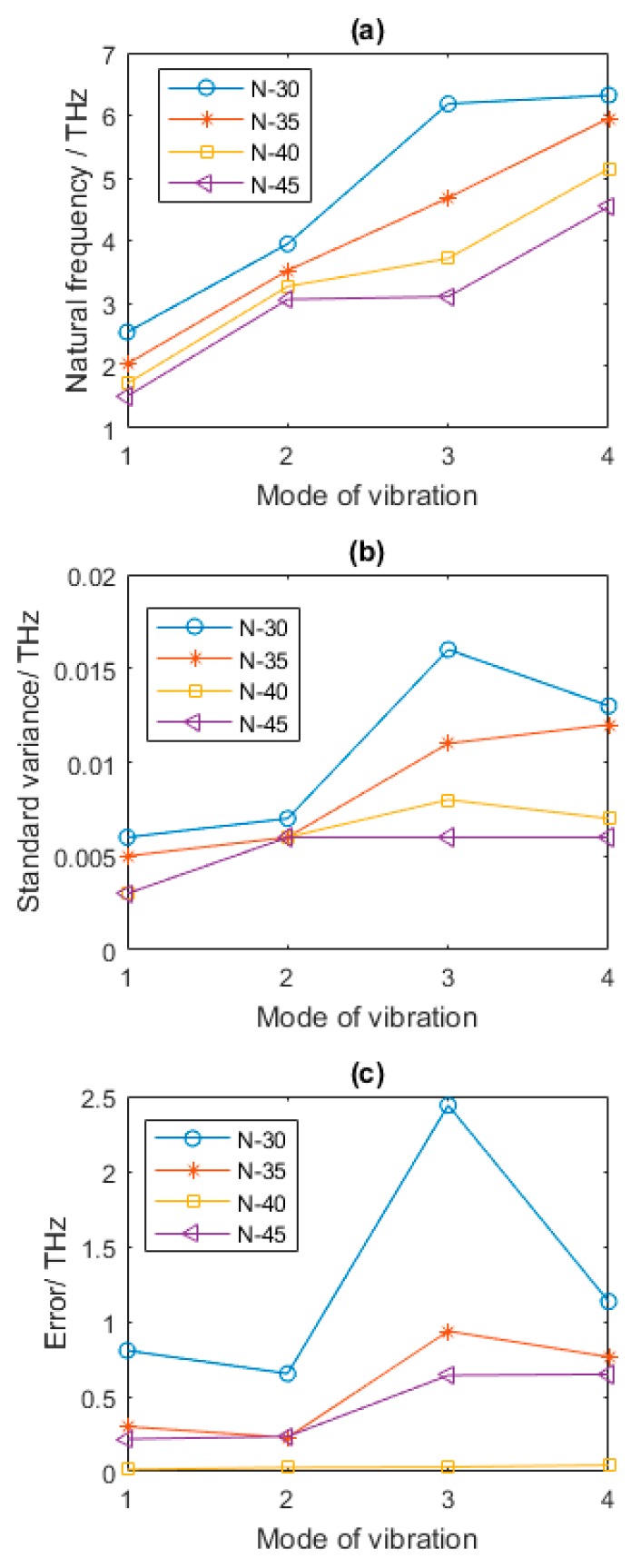
Statistical results of defected graphene sheets with different *N* (**a**–**c** for mean, standard variance and Error of natural frequency, respectively).

There is another geometrical parameter, *N*, the amount of hexagons in *y* axes, which is not only related to the width of graphene sheets, but also corresponding with the amount of vacancy defects if Per is determined as a certain value. Compared with the results in Table 2 and Figure 8, when Per equals to 1% and *N* is 30, the natural frequencies of the first four orders of vibration mode are 47.90%, 20.83%, 66.55%, and 22.85% larger, respectively. When *N* is equal to 35, the increase of the natural frequencies of the first four vibration modes compared with that of graphene in Table 2 are 18.32%, 7.81%, 26.04%, and 15.68%, respectively. When *N* is 45, the reductions of the natural frequencies of the first four order vibration modes are 11.84%, 6.34%, 16.43%, and 11.76%, respectively.

In short, different with the effects of bond length L and the diameter of section cross *D*, the influence of *N* in natural frequencies of vacancy defected graphene sheets is more intricate and disproportionate, as in Figure 8b,c. In general, more emphasis is put on the bond length and the diameter of the cross section, the total width and length of graphene sheets are controlled and limited by experimental operation and chemical methods. However, the number of hexagons in the axes directions is another sensitive factor related to natural frequencies of vacancy defected graphene sheets.

### 4.3. Material Parameters

A large deviation between simulations and experiments in material properties has been found because of the presence of defects in the nanotube structure [2,44]. The mechanical properties of defect-free carbon nanomaterials have not been exactly measured yet. For discussion, material parameters (Young’s modulus and Poisson ratio of graphene sheets) are supposed as specific values.

**Figure 9:**
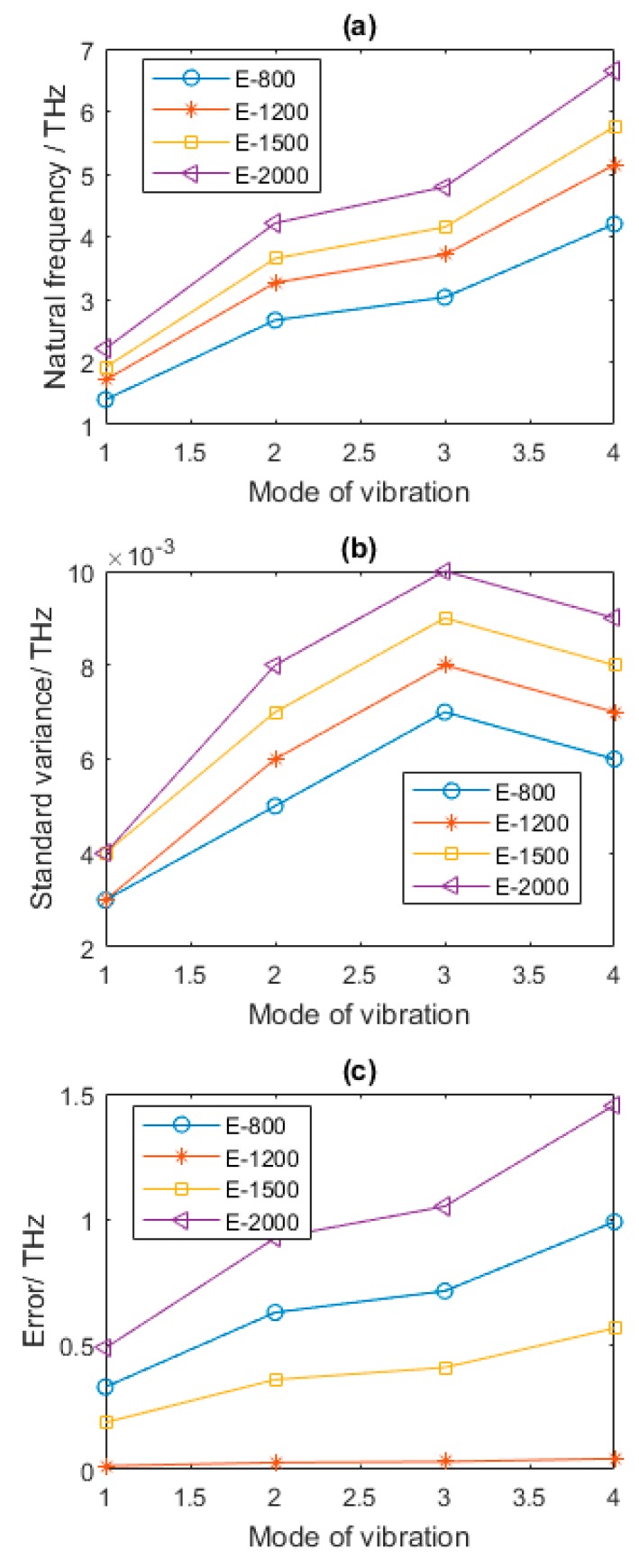
Statistical results of defected graphene sheets with different Young’s modulus (**a**–**c** for mean, standard variance and Error of natural frequency, respectively). *E* is Young’s modulus of graphene sheets.

From Figure 9, it is distinct to find that, with the increase of *E*, namely Young’s modulus, the mean of the first four natural frequencies evidently increased as well. The standard variance and error in Figure 9b also follow this rule. However, the results in Figure 8c are more complicated. When the amount of the vacancy defects is fixed to 1%, and *E* is 800 GPa, the reduction of the natural frequencies of the first four vibration modes compared with that of graphene sheets in Table 2 are 18.38%, 18.35%, 18.34%, and 18.36%, respectively. When *E* is equal to 1500 GPa, the increase of the natural frequencies of the first four vibration modes compared with that of pristine graphene are 11.79%, 11.82%, 11.80%, and 11.80%, respectively. When *E* is 2000 GPa, the increments of the natural frequencies of the first four vibration modes are 29.11%, 29.13%, 29.11%, and 29.09%, respectively.

With the augment of Young’s modulus, the values in stiffness matrix of vacancy defected graphene sheets are amplified, while the stiffness matrix is acted as the numerator, which is reasonable to find the enlargement of natural frequencies in vibration modes. Furthermore, as mentioned in the above results, the first four natural frequencies of graphene sheets all have the close magnitude of change. The effects of Young’s modulus in natural frequencies are convergent to the specific percentage in the different vibration modes.

**Figure 10:**
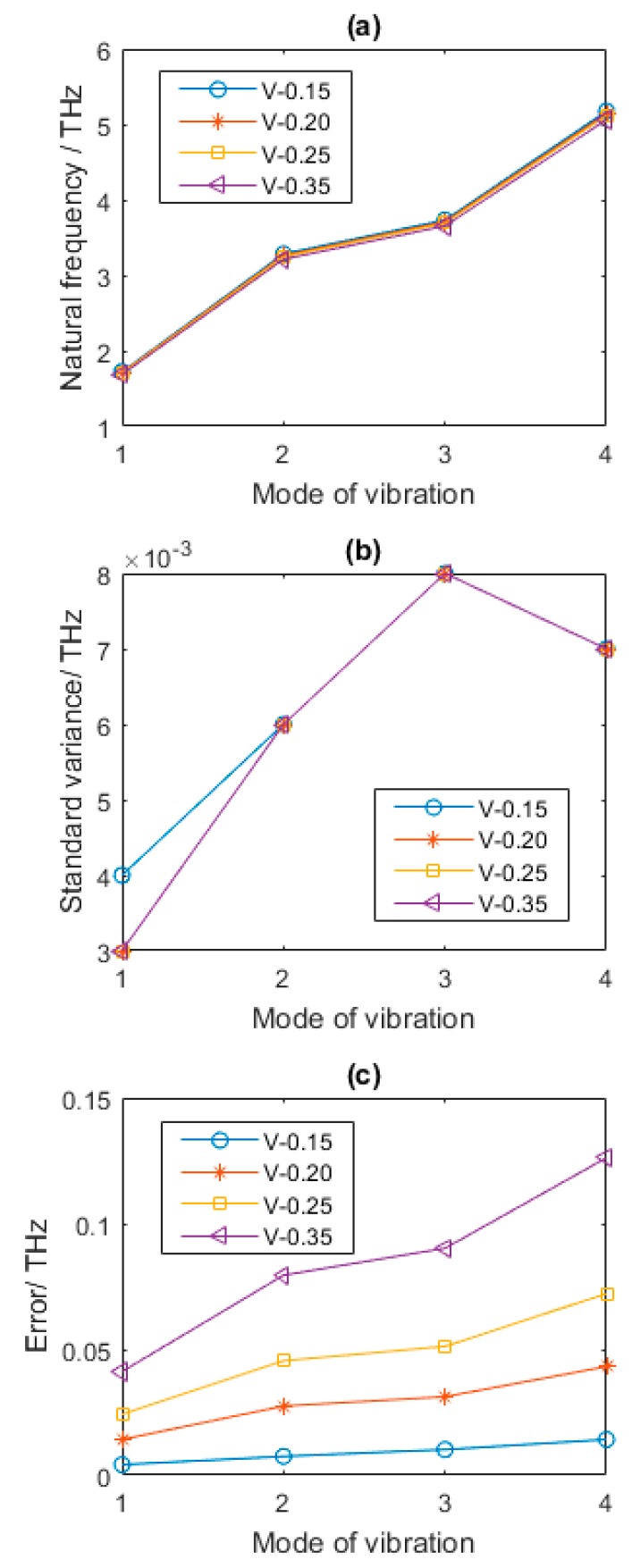
Statistical results of defected graphene sheets with different Poisson ratio (**a**–**c** for mean, standard variance and Error of natural frequency, respectively). *V* is Poisson ratio.

Poisson ratio as one of the parameters corresponding with material properties is discussed in Figure 10. The change of Poisson ratio in vacancy defected graphene sheets does not play significant role in the natural frequencies of graphene sheets. As in Figure 10a, with the increase of *V*, the results of natural frequencies are very approaching. In other words, the Poisson ratio can be simplified as a neglected factor in the analysis of natural frequencies for vacancy defected graphene sheets.

### 4.4. Graphene Sheets with Vacancy Defects

**Figure 11:**
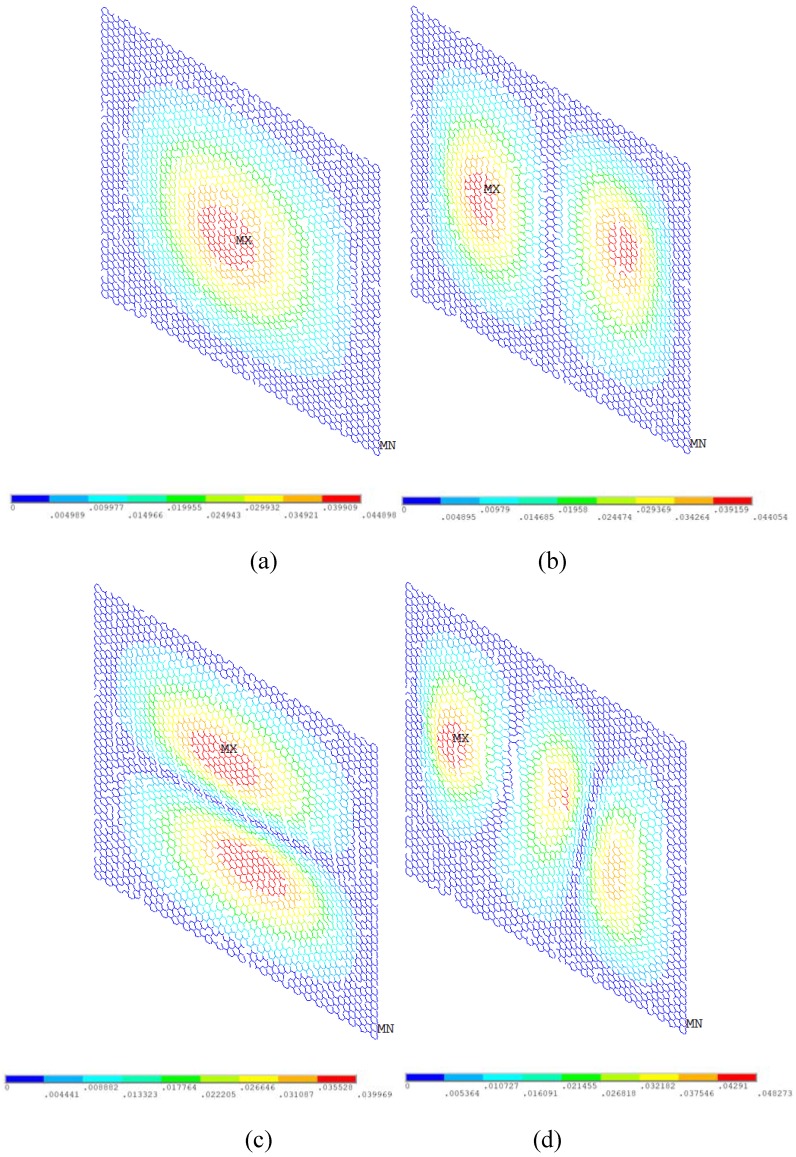
Displacement vector sum for graphene with 5% vacancy defects (**a**–**d** show the first order, second order, third order, and fourth order of vibration mode, respectively).

**Figure 12:**
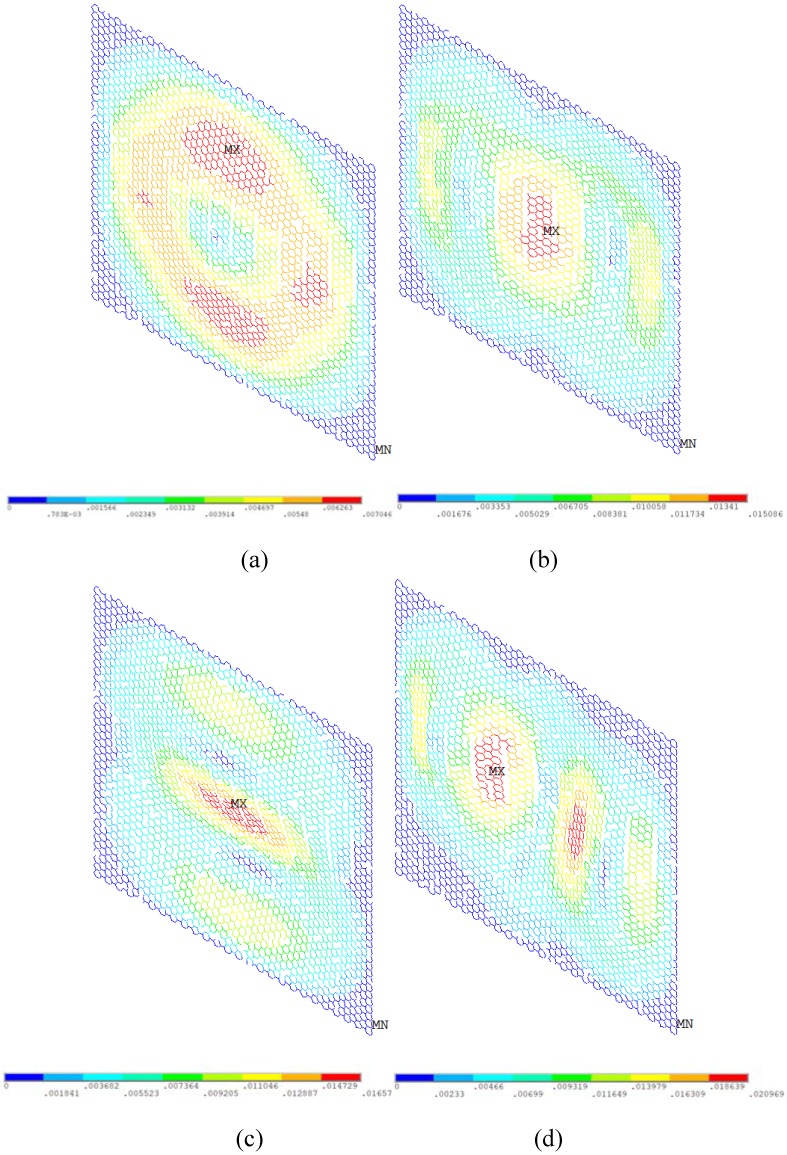
Rotation vector sum for graphene with 5% vacancy defects (**a**–**d** represent the first order, second order, third order, and fourth order of vibration mode, respectively).

In order to observe the vibration behavior of graphene with vacancy defects, Figure 11 and Figure 12 present the displacement vector sum and rotation vector sum for graphene with 5% vacancy defects, respectively. Along with the augment of vacancy defects in the graphene sheet, the natural frequencies of vibration mode are evidently cut down. As demonstrated in Figure 11 and Figure 12, the regular and symmetrical property is also destroyed because of the randomly distributed vacancy defects. Therefore, besides the change of the first four order natural frequencies in the vibration behavior of graphene sheets, the vacancy defects can also deeply influence the displacement and rotation vector of graphene sheets.

In addition, besides the effects of vacancy defects, impurities in graphene sheets are another unavoidable existence and need more attention [45]. Natural frequencies of vacancy defected graphene sheets are a sensitive and appropriate index to comprehensively discuss and study the influence of vacancy defects in stiffness and mass matrices of entire graphene sheets. In addition to mechanical properties, thermal and electronic transport properties are promising in perspective for vacancy defected graphene sheets [46].

## 5. Conclusions

In this paper, an effective Monte Carlo based finite element method to analyze the vibration behavior of vacancy defected graphene sheets is proposed. By combining the Monte Carlo method with the beam FE model, the stochastic dispersed placement of vacancies in graphene is successfully propagated and performed. The results show that,

1. The natural frequencies of vibration decrease dramatically with the increase of the vacancy defects when *Per* is larger than 1%.
2. The regular and symmetrical properties of vibration behavior in displacement and rotation are evidently destroyed when *Per* reaches 5%.
3. Furthermore, with the augment of the vacancy defects quantity, the variances caused by randomly dispersion location of vacancy defects become large.
4. From the discussion about geometrical and material parameters, it can be concluded that the increment of length of each bond in hexagons of graphene sheets distinctly reduces the first four order natural frequencies.
5. For vacancy defected graphene sheets, the amount of hexagons in the long side of the graphene sheet has a similar effect on natural frequencies with length of each bond in hexagon, but it is more complicated.
6. With the increase of thickness of the graphene sheets and Young’s modulus, the natural frequencies of vacancy defected graphene become smaller.

## Figures and Tables

**Table 1:** Comparing the natural frequencies obtained from present model with that in references [26,27,28,29,30,31,32,33,34].

Reference	Simulation Method	Elastic Modulus (TPa)	Poisson’s Ratio	1/THz	2/THz	3/THz	4/THz
Liu [26]	DFT	1.050	0.186	1.6081	3.7232	4.3172	6.4323
Kudin [27]	DFT	1.029	0.149	1.5818	3.6623	4.2466	6.3271
Gupta [28]	MD	1.272	0.147	1.7581	4.0706	4.7201	7.0325
Lu [29]	MD	0.725	0.398	1.4311	3.3135	3.8422	5.7246
Wei [30]	DFT	1.039	0.169	1.5946	3.6921	4.2811	6.3786
Cadelano [31]	TB	0.931	0.310	1.5649	3.6232	4.2012	6.2595
Reddy [32]	MM	0.669	0.416	1.3869	3.2111	3.7234	5.5475
Zhou [33]	MM	1.167	0.456	1.8716	4.3334	5.0248	7.4865
Khatibi [34]	MD + FDD	1.050	0.170	1.6030	2.4970	2.5980	3.5770
Present	SFEM	1.200	0.200	1.7282	3.2925	3.7442	5.1892

**Table 2:** Statistical results of natural frequencies for Monte Carlo based finite element model

*Per* (%)	Mode	Mean (THz)	Variance^^0.5^	Skewness	Kurtosis
0.5	1	1.721	0.002	−0.852	5.179
	2	3.279	0.004	−0.646	3.751
	3	3.729	0.006	−0.536	3.935
	4	5.168	0.005	−0.620	3.625
1	1	1.714	0.003	−0.446	3.144
	2	3.265	0.006	−0.559	3.558
	3	3.713	0.008	−0.495	3.537
	4	5.146	0.007	−0.308	2.757
3	1	1.602	0.292	−5.313	29.246
	2	3.007	0.547	−5.312	29.240
	3	3.512	0.639	−5.312	29.242
	4	4.730	0.861	−5.312	29.243
5	1	1.172	0.584	−1.488	3.247
	2	1.752	0.871	−1.491	3.270
	3	2.196	1.090	−1.501	3.300
	4	2.429	1.204	−1.509	3.316
